# Radiological Evaluation of Penetration of the Irrigant according to Three Endodontic Irrigation Techniques

**DOI:** 10.1155/2016/3142742

**Published:** 2016-06-28

**Authors:** Said Dhaimy, Sara Imdary, Sara Dhoum, Imane Benkiran, Amal El Ouazzani

**Affiliations:** School of Dentistry of Casablanca, Abou Al Alaa Zahar Street 21100, Mers Sultan, Casablanca, Morocco

## Abstract

*Introduction*. This experimental study is to compare radiographs based on the penetration depth of the irrigant following three final irrigation techniques.* Material and Method*. A sample of sixty teeth with single roots were prepared with stainless steel K files followed by mechanized Ni-Ti files iRace® under irrigation with 2.5% sodium hypochlorite. Radiopaque solution was utilized to measure the penetration depth of the irrigant. Three irrigation techniques were performed during this study: (i) passive irrigation, (ii) manually activated irrigation, and (iii) passive irrigation with an endodontic needle CANAL CLEAN®. Radiographs were performed to measure the length of irrigant penetration in each technique.* Results*. In comparison, passive irrigation with a conventional syringe showed infiltration of the irrigant by an average of 0.682 ± 0.105, whereas the manually activated irrigation technique indicated an average of 0.876 ± 0.066 infiltration. Irrigation with an endodontic syringe showed an average infiltration of 0.910 ± 0.043. The results revealed highly significant difference between the three irrigation techniques (*α* = 5%).* Conclusion*. Adding manual activation to the irrigant improved the result by 20%. This study indicates that passive irrigation with an endodontic needle has proved to be the most effective irrigation technique of the canal system.

## 1. Introduction

The endodontic field has advanced with updated techniques and instrumentation, increasing the success rate of the treatments.

Current practice recommends that success in root canal treatment require the removal of infective and necrotic material from the root canal system. Recent studies of the development of mechanical activation systems with advances in endodontic needle design have resulted in increased infiltration of the canal. These include manual agitation technique, machine-assisted agitation systems, continuous irrigation during instrumentation, sonic activation, and laser activation [1, 2].

Root canal instruments create sufficient space for the ingress of irrigant solutions, increasing the success rate of treatment. The limitations of mechanical instruments are apparent, with the elimination of pulp tissue and bacteria within infected canals requiring additional interventions. Given the complex anatomy of root canals, the accessory canals, lateral canals, the anastomosis, and the apical deltas add more complexity to the root canal anatomy, with the limitations of mechanical systems; further study is required with irrigation to progress current practice [3, 4].

Effective irrigant delivery and the activation of the irrigation solution are prerequisites to root canal disinfection and debris removal, to improve the outcomes of endodontic treatment.

Several irrigation agents have been developed in response to chemical disinfection. However, sodium hypochlorite (NaOCl) remains the irrigant of choice [5–7] due to its antibacterial nature, dissolving both necrotic and organic matter within the smear layer [8]. The Scientific Committee Consensus [6, 8–10] recommends a concentration of 2.5% to 5.25% of sodium hypochlorite, providing adequate balance between disinfection and toxicity.

This experimental study compared and evaluated three irrigation techniques of the canal system: passive irrigation, manually activated irrigation using a cone of gutta with a suitable taper (master cone), and irrigation with an endodontic needle.

A radiopaque solution in conjunction with radiography was utilized to evaluate and compare the infiltration of the canal system.

The question this study responds to is the depth of penetration of the irrigant depending on the final activation technique of the irrigant to reach the apical area. Utilizing existing materials can reach optimal treatment outcomes. This study offers significant methodological advance in this field, thus increasing successful treatments with simplified and reproducible methods.

## 2. Material and Method

Sixty maxillary central incisors samples, freshly extracted, were preserved upon extraction in physiological saline solution. All the samples were used for the irrigation techniques.

Samples that were excluded from this study are tooth affected by the extraction, short roots, roots with open apex, and extraction of more than three months and samples unpreserved as soon as the extraction was made.

An individual operator, specialized in endodontics, was responsible for the canal shaping and disinfection of each sample.

The chemomechanical preparation was performed by stainless steel K-files and mechanized Ni-Ti files with fixed taper (iRace), irrigated with sodium hypochlorite diluted to 2.5% with a 2.5 cc syringe and a 21G (40 mm long and 0.8 mm wide). The working length of each sample has been determined by direct vision, using 10 K-file in the canal until the tip of the instrument appears from the apical foramen. The final files that reached the working length were stainless steel 25 K-file and the Ni-Ti file R2 (25/04).

**Figure 1:**
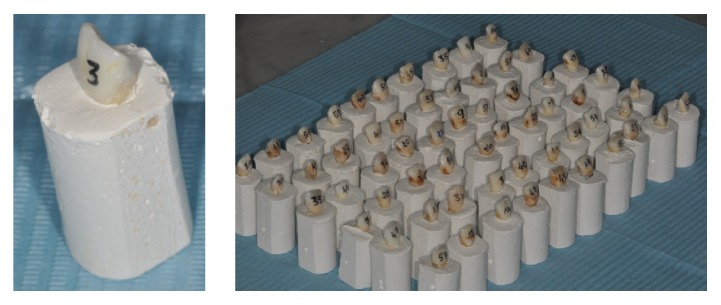
Samples with plinth base.

Each sample was numbered and a layer of varnish applied to the root, to prevent contamination with plaster. Each sample was sunk into the mixture of plaster and sawdust (sawdust increases radiographic contrast) and placed into an individual plinth (Figure 1).

**Figure 2:**
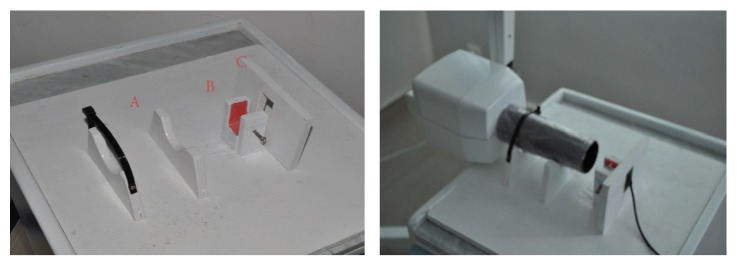
Fixation system. (A) Fixation zone of the radiological cone. (B) Fixation zone of the tooth. (C) Fixation zone of the radiological sensor.

A fixation system was developed and divided into three parts: the first part to fix the X-ray cone (A), the second to place the sample (B), and the third to fix the radiographic sensor (C) (Figure 2).

This fixation system has a major role in this study, because it allowed us to control results. Distances relative to cone/sample and sample/sensor were fixed, thus avoiding potential bias on calculations related to radiation magnification. Radiographic cone was fixed on two slots, perpendicular to the plane of the sensor and to the axis of the sample.

Radiopaque contrast solution (TELEBRIX 35) was applied, allowing visualization of the infiltration. Digital radiography with intraoral sensor was used in conjunction with Kodak Dental Imaging Software 6.12.10.0 to provide immediate results for each technique.

This study was conducted in three stages, each corresponding to three irrigation techniques.

### 2.1. First Stage (Figure 3)

Consider the following:

- Adjustment and fixation of the sample on the fixation system.
- Passive irrigation with radiopaque solution using a 2.5 cc syringe and 21G needle (40 mm long/0.8 mm wide/open ended).
- The needle inserted into the canal until blocked and then retracted 1 mm.
- Irrigation with 2.5 cc of contrast solution using digital pressure.
- A radiograph taken to measure the infiltration of the contrast solution and recorded on Kodak Software.
- The sample irrigated with water to remove the contrast solution and then dried with paper cones.
- A control radiograph taken to confirm the sample preparation for the next stage.

### 2.2. Second Stage (Figure 4)

Consider the following:

- Irrigation with the radiopaque solution using a 2.5 cc syringe and a 21G needle (40 mm long/0.8 mm wide/end open).
- Pumping with gutta master cone into the canal adjusted to the working length minus 1 mm
  - Three push and pull movements.
  - Three pumping motions in three seconds.
  - Amplitude of 5 mm.
- Radiograph taken and recorded.
- Measure of the infiltration of the contrast solution into the root canal recorded on Kodak Software.
- The sample irrigated with water to remove the contrast solution and then dried with paper cones.
- A control radiograph taken to confirm the sample preparation for the next stage.

### 2.3. Third Stage (Figure 5)

Consider the following:

- Irrigation of the sample with the radiopaque solution using an endodontic needle 30G (25 mm length/wide 0.30 mm/side vented) CANAL CLEAN, set to the working length minus 1 mm.
- A radiograph taken to measure the infiltration of the contrast solution and recorded on Kodak Software.

Each set of sixty sample results was separated into three groups, according to the irrigation technique used.

*Group 1.* It contains 60 sample results following irrigation using 2.5 cc syringe with 21G needle (40 mm long/0.8 mm wide/end opened).

*Group 2.* It contains 60 sample results following irrigation using 2.5 cc syringe with 21G needle (40 mm long/0.8 mm wide/end opened) followed by manual activation by three pumping motions of three seconds and 5 mm amplitude using a gutta-percha cone at working length minus 1 mm (master cone).

*Group 3.* It contains 60 sample results following irrigation using endodontic syringe with 30G needle set at working length minus 1 mm (25 mm long/0.30 mm wide/side vented) CANAL CLEAN. 

Radiographic imaging of the samples was assessed with imaging software and the length of the infiltration of the irrigant was measured by drawing a line connecting the coronary landmark to the limit of the infiltration.

An index of infiltration was calculated; the length of the infiltration of the irrigant was divided by the working length:

(1) $$\begin{array}{c} \text{Index of infiltration}=\frac{\text{Infiltration length}}{\text{Working length}}. \end{array}$$

## 3. Results

The data entry of the results and the statistical analysis were made using Epi Info 6.0. The comparison of the average of the three techniques was done using ANOVA Test (analysis of variance).

The ANOVA comparison between group 1 (0.682 ± 0.105) and group 2 (0.876 ± 0.066) has shown that the value of the low standard is 12.270 and the value at 5% threshold is 1.96. This data suggests that there is a* significant difference* between the index of infiltration of the passive irrigation technique and the manually activated irrigation technique using a suitable gutta-percha cone (master cone) with a risk of *α* = 5% (Table 1).

The ANOVA comparison between group 2 (0.876 ± 0.066) and group 3 (0.910 ± 0.043) has shown that the value of the low standard is 3.400 and the value at 5% threshold is 1.96. This data suggests that there is a* highly significant difference* between the index of infiltration of the manually activated irrigation technique using gutta-percha cone and irrigation with the endodontic needle with a risk *α* = 5% (Table 2).

## 4. Discussion

The complexity of the canal anatomy and specifically the apical area has required chemomechanical preparation whereby irrigation with NaOCl of the root canal system has countered the limits of instrumental manoeuvres [6, 11].

It has been accepted that root canal irrigation promotes the removal of 30–50% bacterial biofilm from canal walls without mechanical preparation [12].

Several devices and irrigation techniques have been established to facilitate the root canal debridement. Endodontic needles have allowed appropriate irrigation while respecting the apical area with their lateral slot, flexibility embracing the canal curvatures, and increased control of needle penetration (working length minus 1 mm) resulting in reproducible outcomes.

Three techniques have been the subject of this experiment and were each evaluated by X-ray acquisitions. The first technique was* passive irrigation* with 2.5 cc syringe and 21G needle; the second was* manually activated irrigation technique* by pumping three times with gutta-percha master cone; and the final technique was* irrigation with endodontic syringe* (CANAL CLEAN).

It has been demonstrated that activating irrigants increases their efficiency. Various activation techniques have been developed but can generally be divided into either manual agitation techniques including the use of files and cones of gutta-percha [11–13] or automated agitation devices with sonic and ultrasonic systems [14–18].

For the mechanical preparation of our samples, we used rotary Ni-Ti instruments. Numerous studies have demonstrated their ability to maintain the original curvatures of the canals to provide enough conicity for optimal sealing and to complete the preparation in sufficient time [9, 19–21].

This experimental study was designed to evaluate the infiltration length of the irrigant which is correlated directly with optimal and thorough disinfection of the canal roots.

TELEBRIX 35 contrast solution was the final irrigant to view its penetration into the canal using X-rays. TELEBRIX is a high molecular weight solution and with its decreased fluidity will penetrate slow and shallow into the root canal. In comparison, sodium hypochlorite (NaOCl) has a lower molecular weight solution but increases in infiltration average values can be observed. TELEBRIX also has a radiopaque character enabling the observation of the infiltration directly on the radiographs [19, 22, 23].

The fixation system, radiological tube, sensor, and samples were kept in a standardized position during all the stages of irrigation and X-rays. This prevented expansions and movements during examination.

The results of this study indicate that irrigation using a conventional 21G needle fails to reach optimal depth for a full disinfection objective. Therefore, the technique used by most practitioners does not allow full disinfection of the root canal system which increases the risk of failure of the endodontic therapy.

Evaluation of the irrigant infiltration showed a highly significant difference between passive irrigation and manually activated irrigation. Additionally, a significant difference was observed between the manually activated irrigation and the irrigation with an endodontic needle.

Endodontic needles have shown an improved endodontic irrigation; however, they may be inaccessible to some practitioners. Simple techniques such as the manual activation with suitable tapered gutta indicated a 20% optimization for passive irrigation with 21G needle (40 mm long/0.8 mm wide/open ended).

Methodological studies have been conducted to optimize the chemical disinfection, thus increasing the effect of the irrigant. Assessments of manual and automated agitation systems were focused on the depth of infiltration of the irrigant or its cleaning ability.

The results of this study showed a significant difference between passive and manually activated irrigation with a gutta cone.

Boutsioukis et al. (2007)  [5] suggest that using a gutta cone for hypochlorite activation was not statistically significant compared to passive irrigation. However, this present study used the ProTaper universal gutta cone as a mechanical agitator producing a different result.

Other studies have shown the irrigants infiltration capacity with other activation techniques, such as ultrasonic activation.

Castelo-Baz et al. (2012) [13] have studied the infiltration level of lateral canals. They showed no significant difference in the penetration of sodium hypochlorite in the principal and lateral canals between the ultrasonic irrigation and positive pressure irrigation, while Sabins et al. (2003) [14] demonstrated a highly significant difference between the ultrasonic activation of the irrigant and classic irrigation without activation.

Delivery system of the irrigation solution in the infiltration capacity is crucial; De Gregorio and colleagues (2010) [4] showed that irrigation with negative apical pressure has improved vertical infiltration compared to passive ultrasonic irrigation which has improved horizontal infiltration in lateral canals.

Other studies have investigated the efficacy of disinfection between different irrigation techniques. Mancini and colleagues (2013) [11] compared the ability of removing the smear layer between two activation systems, EndoActivator and EndoVac. It was concluded that passive irrigation with sodium hypochlorite solution or with activation system could remove the smear layer from the canal walls completely. However, EndoActivator and EndoVac showed the best results: 3, 5, and 8 mm from the apex to EndoActivator and 1, 3, 5, and 8 mm for EndoVac.

Root canal disinfection is the essential key to endodontic management. Sodium hypochlorite at 2.5%–5.25% is the irrigant of choice; efficiency is related to the delivery system which must be thin to reach the complex apical regions allowing full disinfection [8, 10, 24]. Considering the complexity of the canal system, full potentiality of the irrigant is required. Utilizing a manual or mechanical activation allows infiltration and a better efficiency. Following the results of this study, we can conclude that manual activation using a taper increased gutta cone permits better infiltration of the irrigant with a significant difference compared to passive irrigation.

## Figures and Tables

**Figure 3:**
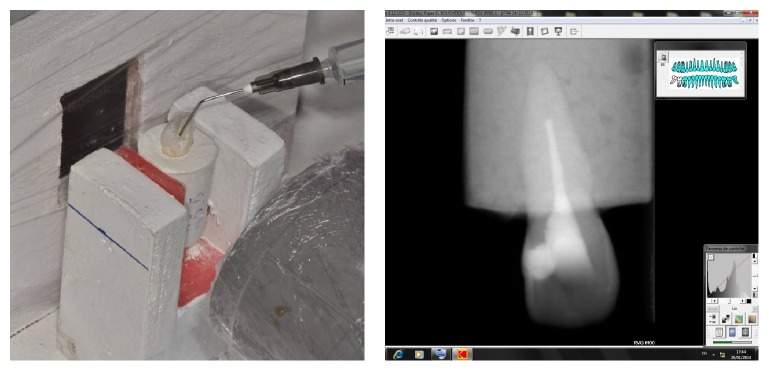
Passive irrigation with conventional needle and the corresponding radiograph.

**Figure 4:**
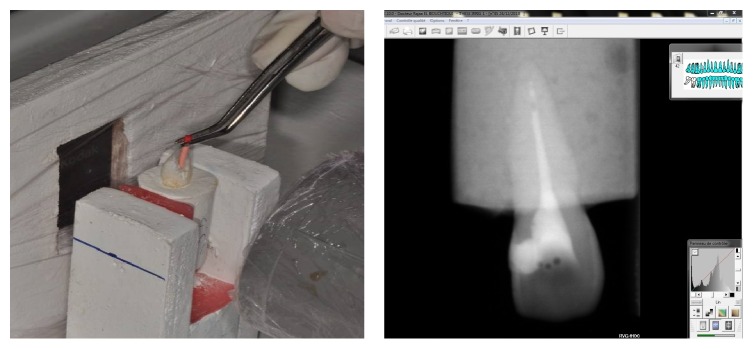
Active irrigation stirring of the cone and the corresponding radiograph.

**Figure 5:**
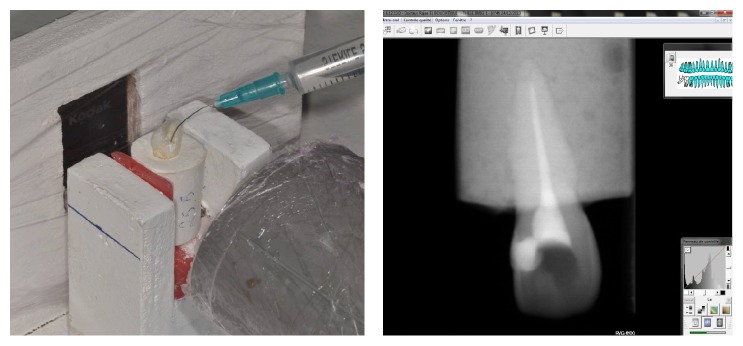
Irrigation with endodontic syringe.

**Table 1:** Comparative analysis of average values of infiltration between group 1 and group 2.

	Average	Variance	Standard deviation
*Group 1* Passive irrigation	0.682	0.011	0.105

*Group 2* Manually activated irrigation	0.876	0.004	0.066

*Low standard* **(**ANOVA Test)			**12.270**

**Table 2:** Comparative analysis of average values of infiltration between group 2 and group 3.

	Average	Variance	Standard deviation
*Group 2* Passive irrigation	0.876	0.004	0.066

*Group 3* Manually activated irrigation	0.910	0.002	0.043

*Low standard* **(**ANOVA Test)			**3.400**
